# Simultaneous suppression of PKM2 and PHGDH elicits synergistic anti-cancer effect in NSCLC

**DOI:** 10.3389/fphar.2023.1200538

**Published:** 2023-05-22

**Authors:** Kaixuan Wang, Hao Lu, Xinmiao Wang, Qingxia Liu, Jinxia Hu, Yao Liu, Meihua Jin, Dexin Kong

**Affiliations:** ^1^ Tianjin Key Laboratory on Technologies Enabling Development of Clinical Therapeutics and Diagnostics, School of Pharmacy, Key Laboratory of Immune Microenvironment and Diseases (Ministry of Education), Tianjin Medical University, Tianjin, China; ^2^ Department of Otorhinolaryngology Head and Neck Surgery, Tianjin First Central Hospital, Tianjin, China; ^3^ Institute of Otolaryngology of Tianjin, Tianjin, China

**Keywords:** PKM2, PHGDH, NSCLC, serine, glycolysis

## Abstract

Metabolic reprogramming is a hallmark of human cancer. Cancer cells exhibit enhanced glycolysis, which allows glycolytic intermediates to be diverted into several other biosynthetic pathways, such as serine synthesis. Here, we explored the anti-cancer effects of the pyruvate kinase (PK) M2 inhibitor PKM2-IN-1 alone or in combination with the phosphoglycerate dehydrogenase (PHGDH) inhibitor NCT-503 in human NSCLC A549 cells *in vitro* and *in vivo*. PKM2-IN-1 inhibited proliferation and induced cell cycle arrest and apoptosis, with increased glycolytic intermediate 3-phosphoglycerate (3-PG) level and PHGDH expression. The combination of PKM2-IN-1 and NCT-503 further suppressed cancer cell proliferation and induced G2/M phase arrest, accompanied by the reduction of ATP, activation of AMPK and inhibition of its downstream mTOR and p70S6K, upregulation of p53 and p21, as well as downregulation of cyclin B1 and cdc2. In addition, combined treatment triggered ROS-dependent apoptosis by affecting the intrinsic Bcl-2/caspase-3/PARP pathway. Moreover, the combination suppressed glucose transporter type 1 (GLUT1) expression. *In vivo*, co-administration of PKM2-IN-1 and NCT-503 significantly inhibited A549 tumor growth. Taken together, PKM2-IN-1 in combination with NCT-503 exhibited remarkable anti-cancer effects through induction of G2/M cell cycle arrest and apoptosis, in which the metabolic stress induced ATP reduction and ROS augmented DNA damage might be involved. These results suggest that the combination of PKM2-IN-1 and NCT-503 might be a potential strategy for the therapy of lung cancer.

## Introduction

Metabolic reprogramming is considered one of the hallmarks of cancer progression (Hanahan and Weinberg, 2011), and may include enhanced aerobic glycolysis, glutaminolysis, amino acid and lipid metabolism, and induction of the pentose phosphate pathway (Schiliro and Firestein, 2021; Zhao and Li, 2021). The Warburg effect, proposed by Otto Warburg in the 1920s, suggests that cancer cells always upregulate glycolysis even in the presence of abundant oxygen (Warburg et al., 1927). This phenomenon is also known as aerobic glycolysis. Glycolysis results in conversion of glucose into pyruvate and generation two molecules of ATP per glucose molecule (Zlacká and Zeman, 2021). Although glycolysis is much less efficient than oxidative phosphorylation (OXPHOS) for generation of ATP (2 *vs.* 36 ATP molecules) (Schiliro and Firestein, 2021; Chae and Hong, 2022), the rate of glycolysis and the turnover of glucose into lactate is much faster than OXPHOS, and therefore results in faster and greater ATP production (Ganapathy-Kanniappan and Geschwind, 2013). In addition, elevated glycolysis is not just for ATP generation, but also supports the rapid growth and proliferation of cancer cells by providing further metabolic intermediates and precursors that are needed to synthesize building blocks, such as amino acids, nucleotides, and lipids (Ganapathy-Kanniappan and Geschwind, 2013; Yaku et al., 2018).

Pyruvate kinase (PK) is a rate-limiting enzyme that catalyzes the last irreversible step of glycolysis, converting phosphoenolpyruvate (PEP) to pyruvate and producing ATP (Israelsen and Vander Heiden, 2015; Dayton et al., 2016). Four pyruvate kinase isoforms are found in mammalian cells, including PKM1, PKM2, PKR, and PKL, which are encoded by two separate genes *PKLR* and *PKM* (Dayton et al., 2016). The expression of PK isoforms is tissue specific. PKM2 is predominantly upregulated in most cancer cells and plays a pivotal role in cancer metabolism and tumor growth (Zahra et al., 2020). PKM2 exists as an inactive monomer, less active dimer, and active tetramer (Wong et al., 2015).

The serine synthesis pathway (SSP) represents an important turning point in glucose conversion (Amelio et al., 2014). Serine is a major one-carbon donor to the folate cycle through one-carbon metabolism, and contributes to nucleotide synthesis, methylation reactions and the generation of NADPH for antioxidant defense to support cell proliferation and combat oxidative stress (Yang and Vousden, 2016). Therefore, serine serves a crucial role in cellular metabolism. Serine is a nonessential amino acid in that its main source is *de novo* biosynthesis. *De novo* SSP consists of three steps, involving the conversion of 3-phosphoglycerate (3-PG, a glycolytic intermediate) to serine through reactions catalyzed by phosphoglycerate dehydrogenase (PHGDH), phosphoserine aminotransferase 1 (PSAT1), and phosphoserine phosphatase (PSPH) (Shuvalov et al., 2017; Schiliro and Firestein, 2021). Serine synthesis is accelerated under metabolic stress, such as glucose and glutamine depletion, and starvation of serine itself (Yang and Vousden, 2016; Schiliro and Firestein, 2021). PKM2 is also regulated by serine, which can bind to and activate PKM2 (Chaneton et al., 2012).

PHGDH catalyzes the rate-limiting first step in the SSP and thus diverts the glycolytic flux into serine biosynthesis (Amelio et al., 2014; Buqué et al., 2021). Amplification of the *PHGDH* gene was identified in several cancers including breast cancer and melanoma, leading to corresponding enzyme overexpression, and decreased PHGDH expression impaired flux into serine synthesis and cell proliferation (Locasale et al., 2011; Mullarky et al., 2011; Possemato et al., 2011). Zhu et al. observed that PHGDH protein in non-small cell lung cancer (NSCLC) tissue was significantly increased compared to matched adjacent lung tissue, and the high level expression of PHGDH positively associated with TNM stage and lymph node invasion (Zhu et al., 2016).

Lung cancer is a public health problem of global magnitude, classified in two main groups: small-cell lung cancer (SCLC) (15% of total diagnoses) and NSCLC (85% of total diagnoses) (Thai et al., 2021). The tumor suppressor liver kinase B1 (LKB1) is frequently mutated (loss-of-function mutations) and inactivated in NSCLC (Gao et al., 2011). LKB1 has been implicated in glucose metabolism through its regulation of AMP-activated protein kinase (AMPK) (Gao et al., 2011). LKB1, also known as serine/threonine kinase 11 (*STK11*), directly phosphorylates AMPK, thus is the activator of AMPK (Luo et al., 2010). AMPK negatively regulates aerobic glycolysis and cellular biosynthesis in cancer cells and inhibits tumor growth (Faubert et al., 2013). Therefore, in the present study we used NSCLC A549 cells (with *LKB1* mutation) to investigate whether simultaneous inhibition of glycolysis and serine biosynthesis synergistically suppresses tumor growth.

Shikonin and its enantiomeric isomer alkannin are reported to be PKM2 inhibitors with similar potency (Chen et al., 2011). Another PKM2 inhibitor, PKM2-IN-1 (also known as Compound 3k) displays more potent PKM2 inhibitory activity than shikonin (Ning et al., 2017), therefore, we used PKM2-IN-1 in our study. PKM2-IN-1 markedly suppressed glycolytic function and cell proliferation in human ovarian adenocarcinoma SK-OV-3 cells, and attenuated tumor growth and mass without significant body weight changes in tumor xenograft mice (Park et al., 2021). However, the anti-cancer effect of PKM2-IN-1 alone or combined with PHGDH inhibitor in NSCLC has not yet been reported.

Here, to capitalize on the link between PKM2 and serine biosynthesis pathway in NSCLC, we used the PKM2 inhibitor (PKM2-IN-1) and PHGDH inhibitor (NCT-503) to evaluate the anti-cancer effect and the mechanism of PKM2-IN-1 in combination with NCT-503 in A549 cells and a xenograft model. We demonstrate that PKM2-IN-1 synergized with NCT-503 to inhibit lung tumor growth *in vitro* and *in vivo*.

## Materials and methods

### Cell culture

Human non-small cell lung cancer A549 cells were obtained from the Cell Resource Center of Peking Union Medical College (Beijing, China). This cell line has been authenticated using STR profiling within the last 3 years. Briefly, A549 cells were cultured in RPMI 1640 medium containing 10% fetal bovine serum (FBS), penicillin (100 U/ml), and streptomycin (100 μg/ml). The cell cultures were maintained in a humidified atmosphere containing 5% CO_2_ at 37°C.

### Reagents

PKM2-IN-1 and NCT-503 were purchased from MedChemExpress (Princeton, NJ, United States). RPMI 1640 and FBS were purchased from Biological Industries (Beit Haemek, Israel). Propidium iodide (PI) was obtained from Sigma-Aldrich (St. Louis, MO, United States). The enhanced chemiluminescence (ECL) reagent was purchased from Thermo Fisher Scientific (Waltham, MA, United States). Antibodies against cyclin B1, cdc2, caspase 3, poly (ADP-ribose) polymerase (PARP), Bax, B-cell lymphoma 2 protein (Bcl-2), pyruvate kinase (PK) M2, phospho-AMP-activated protein kinase (AMPK)α (Thr172), phospho-mTOR (Ser2448), phospho-p70 ribosomal protein S6 Kinase (p70S6K) (Thr389), phospho-histone H2AX (Ser139) (γ-H2AX), p-ATM (Ser 1981), β-actin, and HRP-conjugated goat anti-rabbit and horse anti-mouse secondary antibodies were purchased from Cell Signaling Technology Inc. (Danvers, MA, United States). The anti-p-Chk2 (Thr68) antibody was purchased from Abcam (Cambridge, UK). An antibody specific for PHGDH was purchased from Absin Bioscience Inc. (Shanghai, China). Antibodies against p-p53 (ser15), p53, p21 and Ki67 were purchased from Proteintech Group, Inc (Rosemont, IL, United States). Antibodies against glucose transporter type 1 (GLUT1) was purchased from Abclonal (Woburn, MA, United States).

### Determination of cell viability

Cell viability was assessed using the 3-(4,5-dimethylthiazol-2-yl)-2,5-diphenyltetrazolium bromide (MTT) assay. Briefly, A549 cells were plated in 96-well plates at a density of 1.5 × 10^4^ cells/well and treated with various concentrations of PKM2-IN-1 and/or NCT-503 for 72 h, and then 20 μl of MTT (5 mg/ml) was added to each well. After 4 h of incubation, the formazan was dissolved in DMSO, and the optical density (OD) at 490 nm was measured using an iMark microplate reader (Bio-Rad, Hercules, CA, United States).

### Flow cytometric analysis

A cell apoptosis assay was processed using the Annexin V/FITC-PI Apoptosis Detection Kit (Meilunbio, Dalian, China). Briefly, A549 cells were plated in 6-well plates at a density of 2 × 10^4^ cells/well and treated with PKM2-IN-1 and/or NCT-503 for 72 h. The drug-pretreated cells were harvested with trypsin, resuspended, and incubated with Annexin V-FITC/PI mixture in the dark for 15 min. Subsequently, a total of 1 × 10^4^ cells were collected for each sample and the cell apoptosis was analyzed with BD Accuri C6 flow cytometer (BD Biosciences, San Jose, CA, United States).

For cell cycle analysis, the cells were harvested with trypsin, washed with PBS, fixed with 75% pre-chilled ethanol, and stored at 4°C overnight. After 24 h, the cells were stained with PI. Cells (2 × 10^4^) were then collected for each sample and the cell cycle distribution was analyzed using the BD Accuri C6 flow cytometer.

### Western blotting

Western blot analysis was performed as previously described (Fu et al., 2022). Briefly, cells were lysed in RIPA lysis buffer, and the protein concentration of each sample was determined using a BCA protein assay kit. Protein extracts were separated by 10% sodium dodecyl sulfate-polyacrylamide gel electrophoresis (SDS-PAGE) and transferred to a PVDF membrane. Membranes were blocked with 5% non-fat milk, incubated with primary antibodies at 4°C overnight, washed four times with Tris-buffered saline (TBS) containing Tween-20 (TBST), and incubated with the respective HRP-conjugated secondary antibody for 1 h. Protein bands were visualized using ECL reagents on a Bio-Rad ChemiDoc™ XRS + system (Bio-Rad, Hercules, CA, United States).

### Cellular thermal shift assays (CETSA)

A549 cells were treated with or without PKM2-IN-1 (0.3 μM) for 4 h, then the cells were collected, washed with PBS, and resuspended in PBS containing protease inhibitor cocktail. Each cell suspension was divided into five aliquots respectively, heated at different temperatures (53°C, 56°C, 59°C, 62°C, and 65°C) for 3 min followed by cooling for 3 min at room temperature. The cells were then repeatedly freeze-thawed for three times with liquid nitrogen. Subsequently, every aliquot was centrifuged and then analyzed by Western blotting.

### Measurement of cellular ATP, lactate, and 3-phosphoglycerate (PG)

A total of 4 × 10^4^ A549 cells/well were plated in 6-well dishes and treated with different concentrations of PKM2-IN-1 for 24 h. The cells were then lysed, and the supernatant was collected. The cellular ATP level was determined by using an ATP assay kit (Beyotime, Shanghai, China), following the manufacturer’s instructions. The luminescence signal was measured immediately on a Synergy HTX multimode reader (BioTek Instruments, Inc., Vermont, United States). The lactate and 3-PG levels were analyzed using according to the manufacturers’ instructions for the CheKine™ lactate assay kit (Abbkine, California, United States) and human 3-PG ELISA kit (Keshun Science and Technology Corporation, Shanghai, China), respectively.

### 5-Ethynyl-20-deoxyuridine (EdU) cell proliferation assay

Cell proliferation assays were implemented using BeyoClick™ EdU Cell Proliferation Kit with Alexa Fluor 488 (Beyotime, China) according to the manufacturer’s instructions. Briefly, A549 cells were seeded in 12-well plates and then treated with PKM2-IN-1 and/or NCT-503 for 72 h. The cells were then incubated with the EdU staining buffer for 2 h, fixed with 4% paraformaldehyde for 15 min, and permeabilized with 0.3% Triton X-100 for another 15 min. The cells were incubated with the Click Reaction Mixture for 30 min in a dark place and then incubated with Hoechst 33342 for 10 min. The fluorescence images of EdU incorporation were observed under the Olympus BX51 microscope (Olympus, Tokyo, Japan) and photographed.

### Colony formation assay

A549 cells were seeded onto 6-well plates at a density of 400 cells/well and then treated with PKM2-IN-1 and/or NCT-503 the next day. The culture medium was replaced every 3 days. After incubation for 12 days, colonies were fixed with 4% paraformaldehyde, and then stained with 0.25% crystal violet (Sigma-Aldrich, St. Louis, MO, United States) for visualization. Colonies were counted using ImageJ software.

### Measurement of intracellular ROS levels

Intracellular reactive oxygen species (ROS) levels were determined as described by us previously (Zhao et al., 2021), and detected using the ROS assay kit (Beyotime Biotechnology, China). Briefly, A549 cells were seeded into six-well plates and treated with PKM2-IN-1 and/or NCT-503 for 24 h. Following the treatment, the cells were harvested and then incubated with 10 μM of 2′,7′-dichlorofluorescein diacetate (DCFH-DA) for 30 min at 37°C. After that, the fluorescence was monitored with a flow cytometer.

### Nude mouse xenograft tumor experiments

All animal experiments were approved by and conducted according to the guidelines of the Institutional Animal Care and Use Committee of Tianjin Medical University. Mice were maintained in a pathogen-free environment. A549 cells were injected subcutaneously (s.c.) into the right side of the back of six-week-old male BALB/c nude mice. The sufficiently grown tumor masses were divided into equal pieces and implanted s. c. into the right flank of mice. Mice were randomized into four groups when tumors reached around 70–100 mm^3^ in size, and then PKM2-IN-1 (3 mg/kg) or NCT-503 (30 mg/kg) alone or in combination were administered by intraperitoneal injection every other day for 15 days. The implanted tumor growth was assessed every other day by caliper measurement and calculated using the following formula V = 1/2 × Length × Width^2^.

### Hematoxylin and eosin (H&E) and immunohistochemistry (IHC) staining

H&E and IHC staining were performed as previously reported (Fu et al., 2022). At the end of the experimental period, the tumor, spleen, liver, kidney, lung and heart tissue were extracted when mice were euthanized and fixed in paraformaldehyde and embedded in paraffin for H&E and IHC staining. Tumors were sectioned at 5-μm thickness and stained with H&E for morphological assessment. For immunohistochemistry, slices were deparaffinized, underwent antigen retrieval, and then incubated with antibodies against Ki67 over night at 4°C. Then the sections were further incubated with biotinylated secondary antibodies and diaminobenzidine (DAB). Finally, counterstaining was performed with hematoxylin. Slices were observed under the Olympus BX51 microscope (Olympus, Tokyo, Japan).

### Statistical analysis

All data are expressed as the means ± SD of triplicate values. The statistical data were analyzed by one-way ANOVA and Student’s *t*-test using GraphPad Prism 5 (GraphPad, San Diego, CA, United States). Differences were considered statistically significant at *p* < 0.05.

## Results

### PKM2-IN-1 inhibits cell viability via cell cycle arrest and induction of apoptosis in A549 cells

**FIGURE 1:**
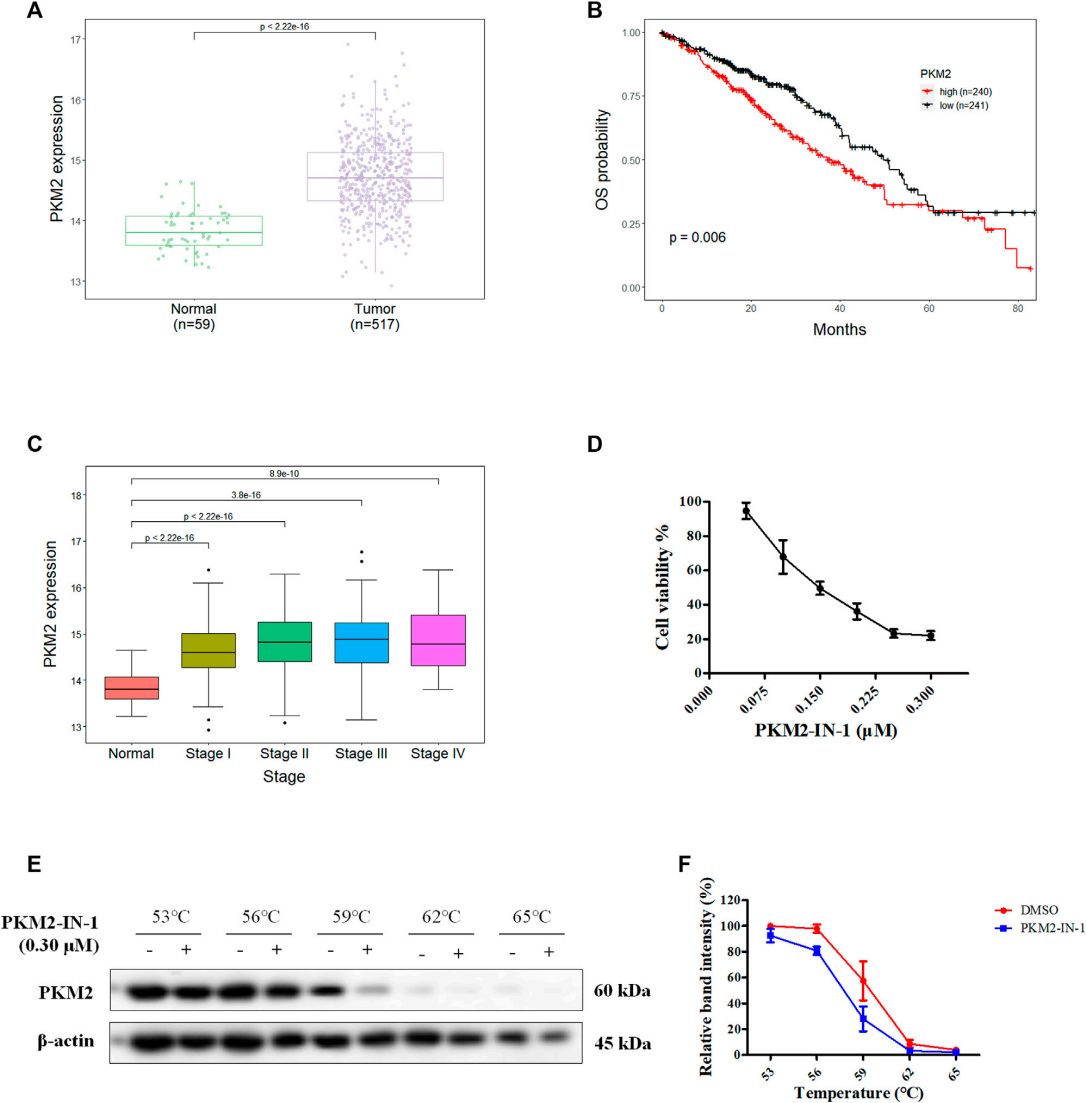
Aberrant PKM2 expression associates with poor prognosis in LUAD. **(A)** Difference in expression of PKM2 between LUAD and normal samples. **(B)** Kaplan-Meier curves for OS of LUAD patients with low and high expression of PKM2. **(C)** PKM2 expression level in normal and four different tumor stage of LUAD samples. **(D)** The effect of PKM2-IN-1 on A549 cell proliferation was determined by MTT assay. **(E)** A549 cells incubated with or without PKM2-IN-1 for 4 h were subjected to CETSA assay. **(F)** CETSA curves of relative band intensity of PKM2. Data are presented as mean ± SD of three independent experiments.

NSCLC are subdivided into adenocarcinoma, squamous cell carcinoma, and large cell carcinoma, and lung adenocarcinoma (LUAD) is the most common subtype (Thai et al., 2021). To investigate PKM2 expression level in LUAD, and association between PKM2 expression and prognosis, we performed bioinformatic analyses of data derived from The Cancer Genome Atlas (TCGA) databases (https://xena.ucsc.edu) using R programming language. We first analyzed the gene expression of PKM2 in 517 LUAD and 59 normal samples from a TCGA dataset and found that PKM2 expression is significantly higher in LUAD tissues than adjacent tissue (*p* < 0.001) (Figure 1A). Moreover, we investigated the correlation between the PKM2 expression and overall survival (OS) of patients with LUAD. The Kaplan-Meier curve showed that the patients with high expression of PKM2 significantly associated with worse OS rates compared to those with low expression of PKM2 (Figure 1B). In addition, we analyzed the relationship between the expression of PKM2 and cancer stages of LUAD patients from TCGA. As shown in Figure 1C, compared to the normal, PKM2 was highly expressed in stages I-IV, which indicates increased expression of PKM2 significantly and positively correlated with increased clinical cancer stages in LUAD patients. Taken together, these results suggest that PKM2 may be an effective biomarker for poor prognosis in LUAD.

To study the anti-cancer biological effect of PKM2-IN-1, we investigated the anti-proliferative effect of PKM2-IN-1 in A549 cells using the MTT assay. A549 cells were treated with various concentrations of PKM2-IN-1 for 72 h. As shown in Figure 1D, PKM2-IN-1 suppressed proliferation of A549 cells in a concentration-dependent manner. The 50% growth-inhibitory concentration (IC_50_) after 72 h treatment to PKM2-IN-1 was 0.15 μM.

The CETSA experiment was used to confirm the interaction of PKM2-IN-1 and PKM2 protein. A549 cells were treated with or without PKM2-IN-1 (0.3 μM), and subjected to CETSA heat pulse. As shown in Figures 1E, F, after treatment with PKM2-IN-1, the level of PKM2 was lower than in the untreated cells. The results revealed that PKM2-IN-1 reduced stabilization of PKM2.

**FIGURE 2:**
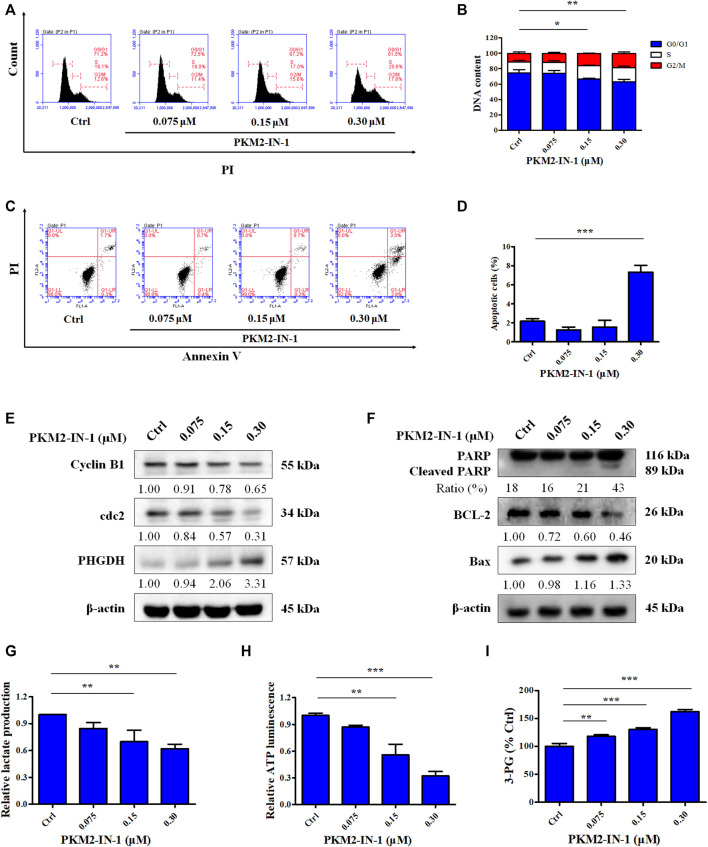
PKM2-IN-1 induces cell cycle arrest and induces apoptosis in A549 cells. **(A)** Cell cycle distribution was detected by flow cytometry after treatment with PKM2-IN-1. **(B)** Statistical analysis of the cell cycle distribution. **(C)** Apoptosis was analyzed by flow cytometry after exposure of A549 cells to PKM2-IN-1. **(D)** Statistical analysis of the cell apoptosis. **(E, F)** The levels of G2/M phase arrest-related and apoptosis-related proteins were measured by Western blotting after treatment with PKM2-IN-1. **(G–I)** Levels of lactate, ATP, and 3-PG were examined after treatment with PKM2-IN-1, respectively. Data are presented as mean ± SD of three independent experiments. *p < 0.05, **p < 0.01 and ***p < 0.001.

To determine the effect of PKM2-IN-1 on the cell cycle progression of A549 cells, flow cytometry analysis was performed on the cells treated with PKM2-IN-1 for 72 h. A549 cells were exposed to 0.075, 0.15, and 0.3 μM of PKM2-IN-1, respectively. Figures 2A, B indicates that PKM2-IN-1 resulted in an increase in the percentage of cell numbers in G2/M phase, accompanied by a decrease of cells in the G0/G1 phase compared with untreated cells. This result indicates that PKM2-IN-1 leads to cell cycle arrest at G2/M phase in a concentration-dependent manner.

Apoptosis is well known to be the major antiproliferative mechanism of anticancer drugs or agents in numerous tumor cell types. To investigate whether the anti-proliferative effect of PKM2-IN-1 was due to induction of apoptosis of NSCLC cells, A549 cells were treated for 72 h with or without PKM2-IN-1 and the pro-apoptotic effect was measured with Annexin V/PI double staining. As shown in Figures 2C, D, PKM2-IN-1 potently increased the apoptotic rate up to 7.4% at a concentration of 0.3 μM. Therefore, based on the results, to examine the mechanism of cell cycle arrest and apoptosis induction of PKM2-IN-1 in A549 cells, we determined the effects of PKM2-IN-1 on the expression of cell cycle regulatory proteins and apoptosis-related proteins by Western blotting. The cyclin-dependent protein kinase (CDK) 1 (also known as cell division cycle protein 2, cdc2) - cyclin B complex is activated in G2 phase, and CDK1-cyclin B complex launches entry and progression into the M phase of the cell cycle (Roskoski, 2019). As shown in Figure 2E, PKM2-IN-1 treatment for 72 h substantially decreased cell cycle related proteins such as cyclin B1 and cdc2 levels in a concentration-dependent manner. The Bcl-2 family of proteins comprises both pro-apoptotic and anti-apoptotic members, which play a crucial role in regulating cell death. PKM2-IN-1 treatment resulted in increased pro-apoptotic Bax and decreased anti-apoptotic protein Bcl-2, as well as increased cleaved PARP levels (Figure 2F). These results indicate that PKM2-IN-1 attenuation of cell proliferation might be mediated by inducing cell cycle arrest and apoptosis.

### PKM2-IN-1 regulates the generation of glycolysis products or intermediates including cellular lactate, ATP, and 3-PG in A549 cells

Dimeric PKM2 regulates the rate-limiting step of glycolysis that shifts glucose metabolism from the normal respiratory chain to lactate production in tumor cells (Zahra et al., 2020). Low enzymatic activity of dimeric PKM2 could divert glucose metabolism towards anabolism through aerobic glycolysis (Wong et al., 2015). To identify the effect of PKM2-IN-1 on glycolysis, the generation of metabolic products or intermediates, such as cellular lactate, ATP levels, and 3-PG in A549 cells, was quantitatively analyzed. Cells were incubated with PKM2-IN-1 for 24 h and production of cellular lactate, ATP, and 3-PG was determined. As shown in Figure 2G, lactate concentration in the cells decreased after PKM2-IN-1 treatment. In addition, PKM2-IN-1 inhibited ATP production in a dose-dependent manner (Figure 2H). However, the level of the glycolytic intermediate 3-PG was enhanced by PKM2-IN-1 treatment (Figure 2I). Moreover, Western blot analysis showed that PKM2-IN-1 treatment resulted in the upregulation of the expression of PHGDH (Figure 2E). PHGDH is a rate-limiting enzyme that converts 3-PG to serine (Yang and Vousden, 2016; Buqué et al., 2021). These findings demonstrate that PKM2-IN-1 reduces glycolysis and accumulation of the glycolytic intermediate 3-PG, and thus might flux into the serine biosynthesis pathway in A549 cells.

### PKM2-IN-1 synergizes with NCT-503 to inhibit A549 cell proliferation

**FIGURE 3:**
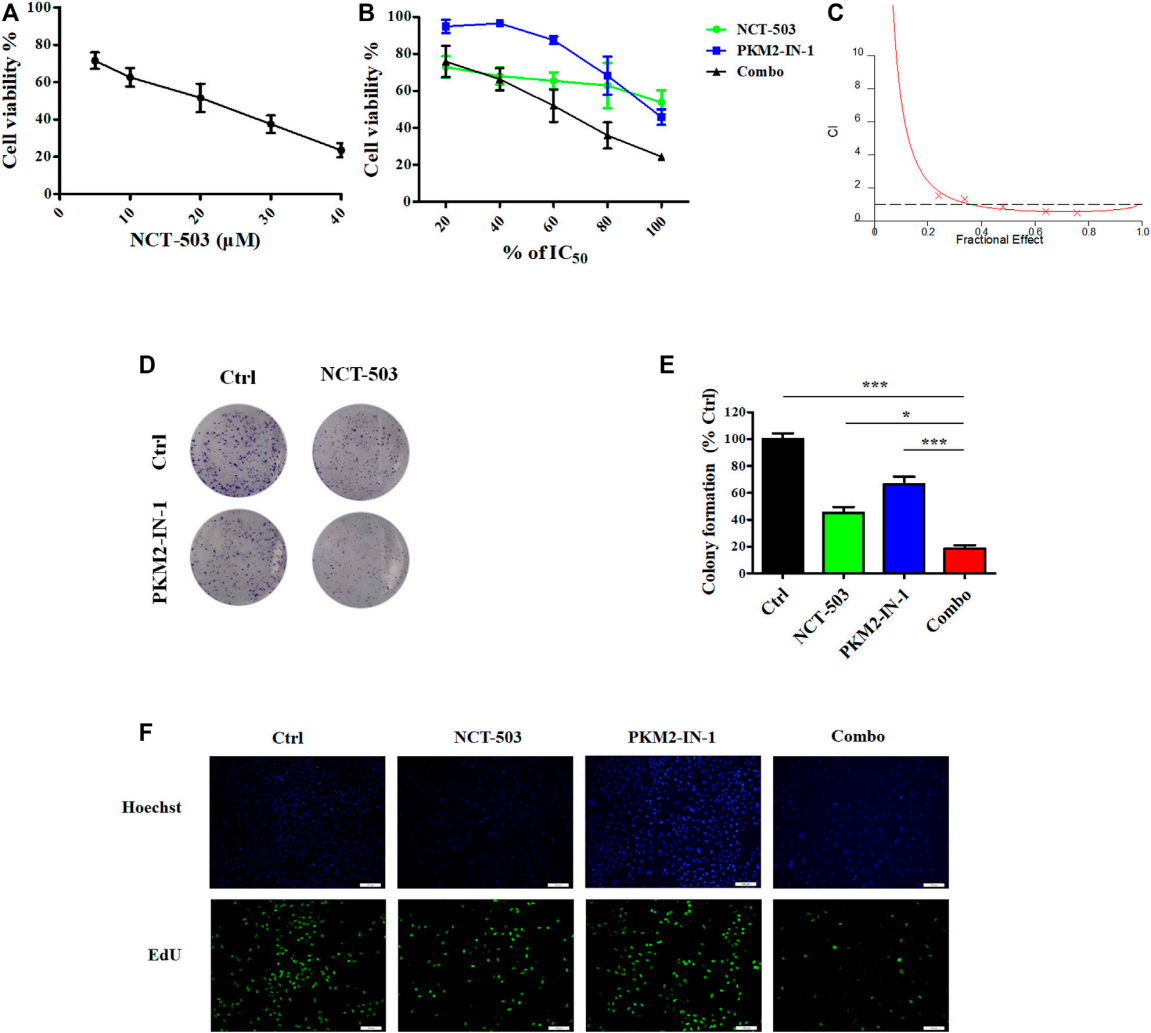
PKM2-IN-1 in combination with NCT-503 inhibits cell proliferation, colony formation, and EdU incorporation. **(A)** After treatment with different concentrations of NCT-503 for 72 h, the cell viability of A549 cells was measured by the MTT assay. **(B)** A549 cells were treated with various percentages of the IC_50_ of PKM2-IN-1 and NCT-503 for 72 h, and then cell viability was analyzed. **(C)** Combination index for A549 cells treated with combined PKM2-IN-1 and NCT-503. **(D)** The cell colonies were stained with crystal violet and observed under an inverted microscope. **(E)** Statistical analysis of the number of cell colonies in PKM2-IN-1, NCT-503, and combination treated cells. **(F)** Fluorescence micrographs of PKM2-IN-1, NCT-503, and combination treated cells with EdU incorporation. Green: EdU-positive cells; Blue: Hoechst for nuclear staining. Data are presented as mean ± SD of three independent experiments. *p < 0.05 and ***p < 0.001.

PHGDH is the key enzyme of *de novo* serine biosynthesis, converting the intermediate of glycolysis 3-PG to produce serine (Shuvalov et al., 2017). Based on the above results, we hypothesized that simultaneously inhibiting PKM2 and PHGDH may more efficiently delay tumor growth. Therefore, we investigated the anti-proliferative effect of NCT-503 using an MTT assay. Figure 3A revealed that NCT-503 treatment effectively suppressed A549 cells proliferation with the IC_50_ value of 16.44 μM for 72 h. To investigate whether PKM2-IN1-1 and NCT-503 have a synergistic anti-cancer effect, the Chou-Talalay method was used to evaluate the combination effect. The MTT assay was performed to measure the inhibitory activities of a series of drug concentration combinations (20, 40, 60, 80, and 100% of IC_50_ values of each drug). Combination index (CI) plots were generated using CalcuSyn software, where CI < 1, = 1, and >1 indicate synergistic, additive, and antagonistic effects respectively. As shown in Figures 3B, C, PKM2-IN1-1 and NCT-503 in combination show synergism with CI values ED_50_, ED_75_, and ED_90_ of 0.68670, 0.58084, and 0.67531, respectively. Therefore, the ratio of concentration IC_50__PKM2-IN1-1_:IC_50__NCT-503_ (0.15 μM:16.5 μM) was fixed for subsequent study.

Furthermore, we investigated the anti-proliferative effects of PKM2-IN1-1 in combination with NCT-503 by colony formation assay, which is widely used to analyze the anti-proliferative effect of drugs. PKM2-IN1-1 and NCT-503 treatment reduced colony formation compared with non-treated cells, and the combination of PKM2-IN1-1 and NCT-503 more remarkably reduced colony formation compared with either drug alone (Figures 3D, E).

In addition, we further analyzed cell proliferation by the EdU staining assay, which has been commonly used to indicate DNA synthesis, to confirm the synergistic anti-proliferation activity of PKM2-IN1-1 in combination with NCT-503. As shown in Figure 3F, the proportion of EdU-positive cells was higher in the non-treated control group, and was significantly reduced by treatment of PKM2-IN1-1 in combination with NCT-503.

### PKM2-IN-1 in combination with NCT-503 causes increased cell cycle arrest in G2/M phase in A549 cells

**FIGURE 4:**
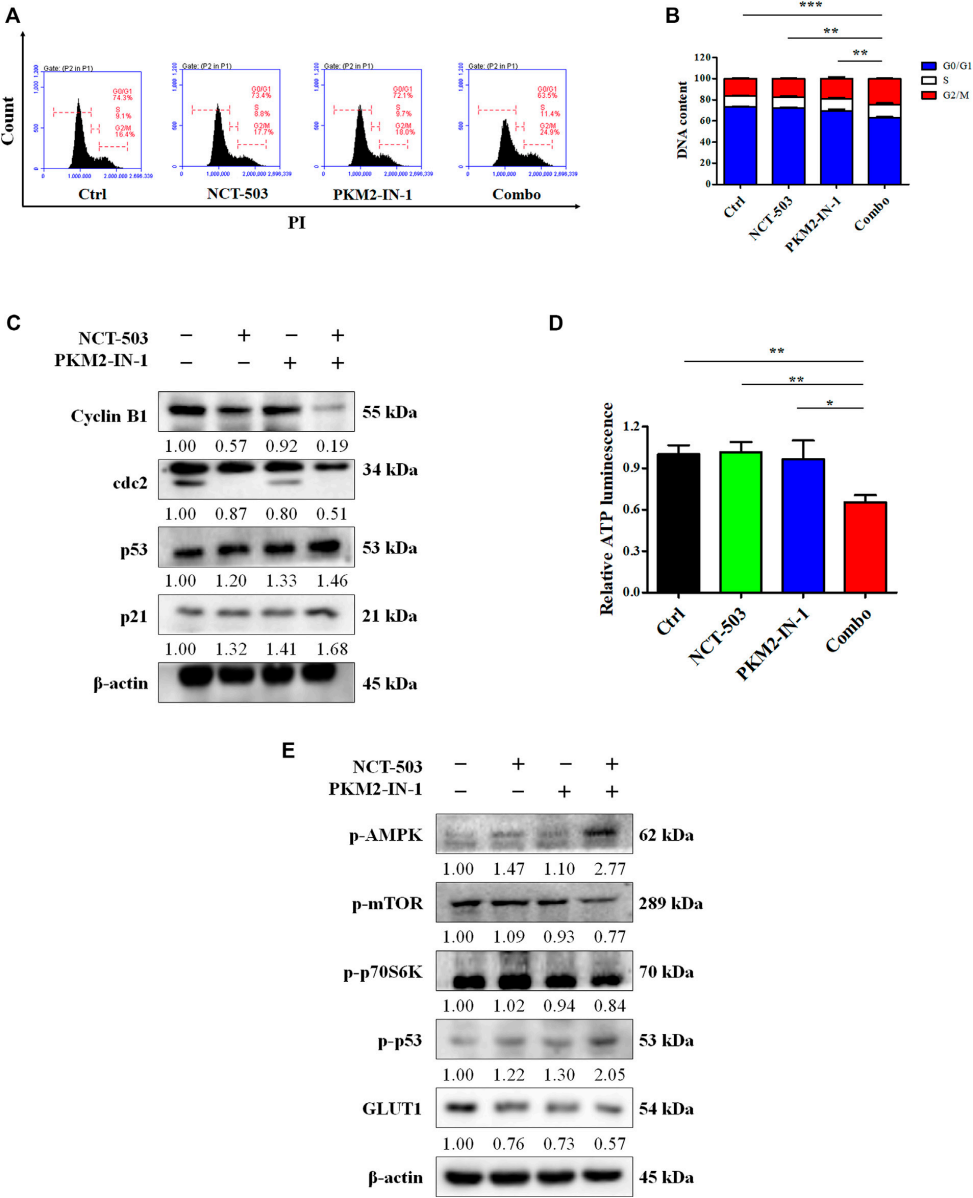
PKM2-IN-1 in combination with NCT-503 induces cell cycle arrest via the AMPK-mediated signaling pathway, and inhibits GLUT1 expression. **(A)** Cell cycle distributions were measured by flow cytometry after treatment with PKM2-IN-1, NCT-503, and a combination of the two agents. **(B)** Statistical analysis of the cell cycle distribution. **(C)** The levels of G2/M phase arrest-related proteins were measured by Western blotting after treatment with PKM2-IN-1, NCT-503, and combination of the two agents. **(D)** Intracellular ATP level of A549 cells was examined after 24 h treatment with combination of PKM2-IN-1 and NCT-503. **(E)** The levels of p-AMPK, p-mTOR, p-p70S6K, p-53, and GLUT1 were determined by Western blotting. *p < 0.05 and **p < 0.01.

Cell cycle regulation is vital for cell proliferation and growth. Therefore, to determine whether the PKM2-IN-1/NCT-503 combination inhibits A549 cells proliferation was due to altered cell cycle distribution, we analyzed the cell cycle under combination treatment by flow cytometry. We found that PKM2-IN1-1 in combination with NCT-503 induced significant G2/M phase arrest compared to either single agent treatment (Figures 4A, B). To investigate the mechanism of combination treatment on cell cycle arrest, the levels of cell cycle-related proteins were examined by Western blotting. p21 is a CDK inhibitor and a transcriptional target of p53 (Taylor and Stark, 2001). Figure 4C shows that combination treatment caused reduced cyclin B1 and cdc2 levels and increased the level of p21. Combination treatment also increased the level of p53.

### Combination of PKM2-IN-1 and NCT-503 regulates AMPK/mTOR and AMPK/p53/GLUT1 pathways in A549 cells

Glucose deprivation leads to cellular ATP depletion and ROS accumulation, which in turn activates AMPK (Zhao et al., 2017). To confirm this, we first investigated the ATP levels in A549 cells treated with PKM2-IN-1 and/or NCT-503. As shown in Figure 4D, the ATP level decreased significantly when cells were exposed to PKM2-IN-1 in combination with NCT-503 compared to PKM2-IN-1 or NCT-503 alone. AMPK activation can suppress the mTOR1 signaling pathway (Wang et al., 2016). Western blot results showed that after A549 cells were treated by PKM2-IN-1 in combination with NCT-503, the p-AMPK level was notably elevated and p-mTOR and p-p70S6K were drastically decreased compared to the PKM2-IN-1 or NCT-503 single agent.

AMPK also induces phosphorylation of p53 on Ser15, which is required for AMPK-regulated cell cycle arrest (Jones et al., 2005). Wild-type p53 has a negative effect on the transcriptional activity of the promoter for the *GLUT1* gene (Schwartzenberg-Bar-Yoseph et al., 2004). We found that the combination of PKM2-IN-1 and NCT-503 resulted in p-p53 (Ser15) upregulation and greater downregulation of GLUT1 expression compared to each treatment alone (Figure 4E).

### PKM2-IN-1 in combination with NCT-503 increases ROS production and DNA damage and results in induction of apoptosis in A549 cells

**FIGURE 5:**
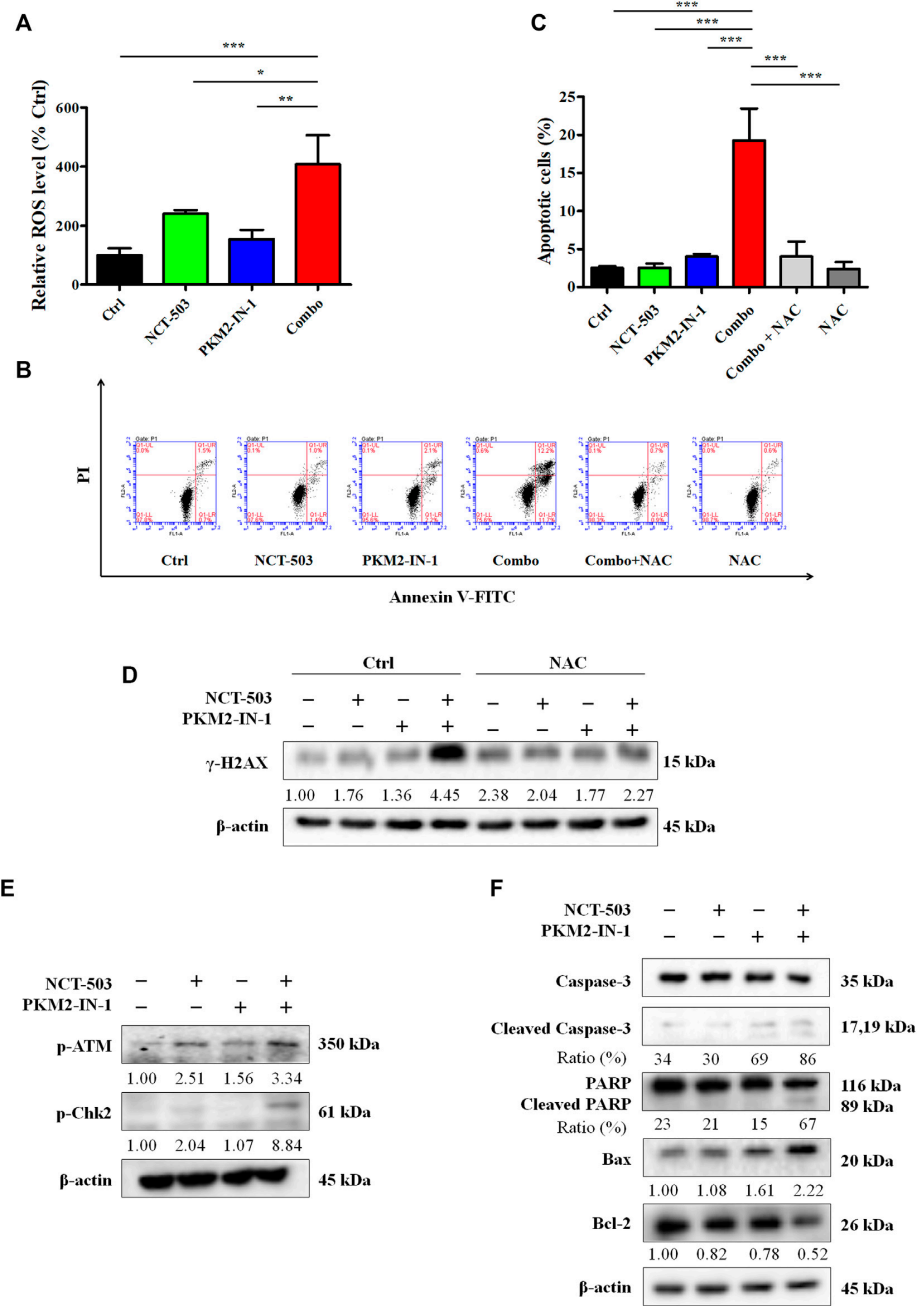
PKM2-IN-1 in combination with NCT-503 induces A549 cells apoptosis by triggering intracellular ROS generation. **(A)** The intracellular ROS level was detected by flow cytometry after treatment with PKM2-IN-1 in combination with NCT-503 for 24 h, and the relative ROS level was calculated. **(B)** The apoptosis of A549 cells was detected after exposure to PKM2-IN-1 in combination with NCT-503 in the presence or absence of NAC for 72 h by flow cytometry. **(C)** Statistical analysis of the cell apoptosis. **(D)** The level of γ-H2AX was assessed in A549 cells exposed to PKM2-IN-1 in combination with NCT-503 in presence or absence of NAC for 72 h by Western blotting. **(E)** DNA damage-related proteins p-ATM and p-Chk2 levels were evaluated by Western blotting. **(F)** Apoptosis-related proteins were evaluated by Western blotting. Data are presented as mean ± SD of three independent experiments. *p < 0.05, **p < 0.01 and ***p < 0.001.

Next, we performed the DCFH-DA flow cytometry assay to analyze the ROS level. Figure 5A shows that PKM2-IN-1 in combination with NCT-503 obviously increased the ROS level compared to untreated and PKM2-IN-1 or NCT-503 alone treated groups. Excess cellular levels of ROS can lead to activation of cell death processes such as apoptosis (Redza-Dutordoir and Averill-Bates, 2016). Therefore, A549 cells were treated with PKM2-IN-1 or NCT-503 alone or in combination for 72 h and cell apoptosis was detected. PKM2-IN-1 in combination with NCT-503 significantly induced apoptosis when compared to PKM2-IN-1 or NCT-503 alone. We also found that the antioxidant N-acetyl cysteine (NAC, a ROS scavenger) obviously inhibited apoptosis induced by PKM2-IN-1 in combination with NCT-503 in A549 cells (Figures 5B, C). ROS can directly or indirectly damage DNA (Renaudin, 2021), and the cellular responses to DNA damage are collectively termed the DNA damage response (DDR) (Li et al., 2016). γ-H2AX (phosphorylated H2AX) is a marker of DDR. Simultaneous treatment with PKM2-IN-1 and NCT-503 A549 cells remarkably elevated γ-H2AX level compared to the untreated or single agent treated cells (Figure 5D), which was alleviated by 2 mM of NAC. As expected, PKM2-IN-1 in combination with NCT-503 treatment dramatically increased levels of the DNA damage-related proteins p-ATM and p-Chk2 compared to the single treatment (Figure 5E). These results indicate that PKM2-IN-1 in combination with NCT-503 induced cell apoptosis was related to ROS triggered DNA damage.

To confirm PKM2-IN-1 in combination with NCT-503 induces the apoptotic mechanism, we determined the levels of apoptotic-related proteins, key molecules mediating apoptotic signal pathway proteins, cleaved caspase-3, and cleaved PARP. As shown in Figure 5F, compared with single agent treatment, cleaved caspase-3 and cleaved PARP were potently increased in A549 cells when treated with combination of PKM2-IN-1 and NCT-503. In addition, combined PKM2-IN-1 and NCT-503 treatment led to upregulated pro-apoptotic Bax expression and downregulated anti-apoptotic Bcl-2 expression.

### Combination of PKM2-IN-1 and NCT-503 suppresses A549 tumor growth in nude mouse xenograft model

**FIGURE 6:**
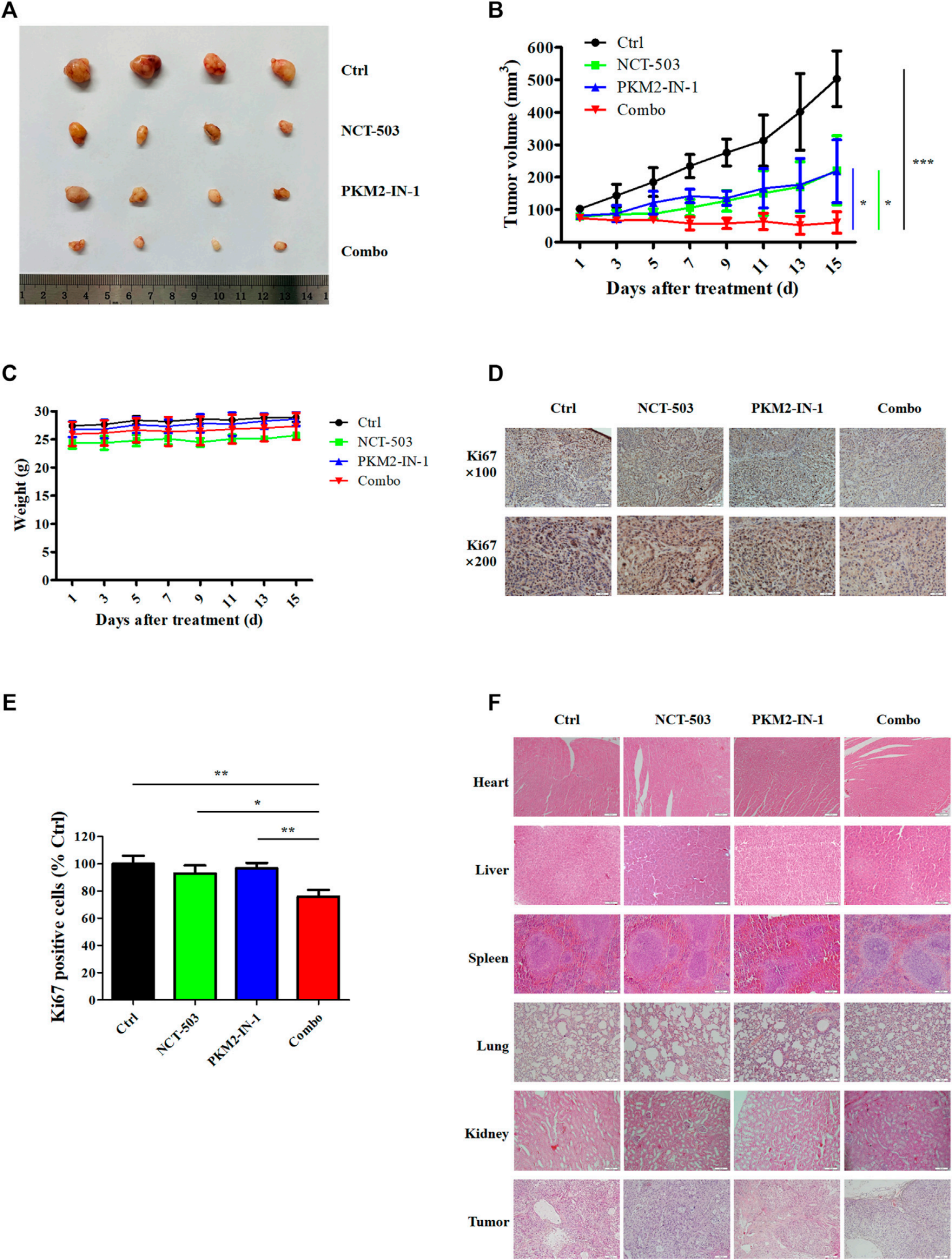
PKM2-IN-1 cooperates with NCT-503 to inhibit tumor growth in A549 tumor nude mouse xenograft models. **(A)** Representative images of the tumors removed from nude mice after treatment with PKM2-IN-1, NCT-503, and combination of two agents. **(B)** The tumor volumes were measured and calculated. **(C)** Measurement of body weights every 2 days. **(D, E)** The expression of Ki67 was revealed by IHC in tumor xenograft tissues. **(F)** H&E staining of different tissues and tumor. *p < 0.05, **p < 0.01 and ***p < 0.001.

To evaluate further the antitumor effects of PKM2-IN-1 alone or in combination with NCT-503 *in vivo*, a nude mice model bearing A549 cell xenografts was constructed. As shown in Figures 6A, B, PKM2-IN-1 attenuated growth of A549 tumor xenografts compared to control (no treatment). PKM2-IN-1 combined with NCT-503 significantly decreased tumor volume compared with PKM2-IN-1 or NCT-503 alone. We found no obvious change in body weight in combination treated mice (Figure 6C). Ki67 is a commonly used marker of cancer cell proliferation. As displayed in Figures 6D, E, we verified that PKM2-IN-1 in combination with NCT-503 treatment led to a weaker Ki67 staining in IHC as compared with the single agent treatment group. Furthermore, H&E staining of the major tissues, such as heart, liver, spleen, lung, and kidney, showed that combination of PKM2-IN-1 and NCT-503 had no apparent distinct organ related toxicities in mice (Figure 6G).

## Discussion

Glycolysis is recognized as the main metabolic pathway in highly proliferative cells such as cancer cells (Zlacká and Zeman, 2021). PKM2 plays an important role in glycolysis and has the ability to transition between the low-activity dimer form and the highly active tetramer form as a glycolytic enzyme (Zahra et al., 2020). The tetrameric PKM2 promotes high ATP production and dimeric PKM2 initiates high rates of biosynthesis (Wong et al., 2015). In tumor cells, PKM2 tends to exist as dimer form to increase anabolic synthesis of macromolecules. In the present study, we investigated the anti-cancer effect of PKM2-IN-1 alone or in combination with NCT-503 both *in vitro* and *in vivo* in NSCLC A549 cells and xenografts. Our data revealed that PKM2-IN-1 significantly inhibited NSCLC A549 cell proliferation in a concentration dependent-manner. The cell cycle consists of the G1, S, G2, and the mitotic (M) phase (Wenzel and Singh, 2018). G2 marks the gap phase between the end of S phase and the start of mitosis (M). During the G2 phase, B-type cyclins are actively synthesized and bind CDK1, and CDK1-cyclin B complexes are essential for initiating mitosis (Diaz-Moralli et al., 2013). Our data indicates that PKM2-IN-1 increases the proportion of cells in the G2/M phase, accompanied by a decrease in the percentage of cells in the G1 phase, while potently down-regulating the expression levels of cyclin B1 and cdc2, resulting in G2/M cell cycle arrest. These results indicate that PKM2-IN-1 reduces cyclin B1 and cdc2 expression, thereby leading to cell cycle arrest in G2 phase without entering the M phase.

Apoptosis is a major form of cell death, characterized by maintenance of membrane integrity until very late in the death process (Cohen, 1997; Kinloch et al., 1999). Apoptosis is divided into extrinsic (death receptor pathway) and intrinsic (mitochondrial pathway) pathways (Ou et al., 2017). Intrinsic stress including direct DNA damage, oncoproteins, hypoxia, and survival factor deprivation, can activate the intrinsic apoptosis pathway (Johnstone et al., 2002). The intrinsic apoptotic pathway is mainly regulated by the Bcl-2 family of proteins, which govern the release of cytochrome C from the mitochondria and activation of caspase-9, stimulating effector caspases, and caspase-3 (Haupt et al., 2003; Kim and Kim, 2018). Caspase-3 is responsible for the proteolytic cleavage of PARP (Cohen, 1997), a critical enzyme involved in DNA repair and many other cellular processes including transcription, replication, and modulation of chromatin structure (Morales et al., 2014). We found that treatment with PKM2-IN-1 significantly increased apoptotic cells compared to untreated control cells, and simultaneously increased Bax, decreased the anti-apoptotic Bcl-2 protein level, and upregulated cleaved PARP. These data suggest that PKM2-IN-1 triggers mitochondria-mediated apoptosis via increasing the Bax/Bcl-2 ratio and increasing cleavage of PARP in A549 cells.

In our study, we found that PKM2-IN-1 treatment reduced lactate and ATP production in A549 cells and potently elevated the intermediate 3-PG. PKM2-IN-1 also significantly increased the expression of PHGDH. These findings imply that PKM2-IN-1 causes the accumulation of the intermediate 3-PG through inhibition of glycolysis, and 3-PG might feed into serine synthesis. Serine is synthesized *de novo* from the glycolytic intermediate 3-PG, which is converted by PHGDH, PSAT1, and PSPH into serine, thus SSP is one side branch of glycolysis (Amelio et al., 2014; Yang and Vousden, 2016). Serine is also an activator of PKM2, and serine depletion reduces PKM2 activity in cells (Chaneton et al., 2012).

Based on above results that PKM2-IN-1 can accumulate 3-PG and upregulate PHGDH, we hypothesized that PKM2-IN-1 in combination with the PHGDH inhibitor may display a synergistic anti-tumor effect. In the present study, we validated that treatment with PKM2-IN-1 and NCT-503 remarkably reduced the proliferation of A549 cells when compared to PKM2-IN-1 or NCT-503 alone. In addition, when cells were treated simultaneously with PKM2-IN-1 and NCT-503, colony formation was potently inhibited and the percentage of proliferating cells, determined by EdU incorporation, was reduced. Consistent with these results, PKM2-IN-1 in combination with NCT-503 treatment increased the G2/M population through downregulation of the protein expression of cyclin B1 and cdc2. p21 as a CDK inhibitor plays a pivotal role in controlling cell cycle progression. p53 induces expression of p21 in response to cellular stress, including oxidative stress and DNA damage (Shamloo and Usluer, 2019). In addition, after treatment with the combination of PKM2-IN-1 and NCT-503, the levels of p53 and p21 increased significantly as compared to the cells treated only with PKM2-IN-1 or NCT-503. These data suggest that PKM2-IN-1 in combination with NCT-503 arrested the cell cycle at the G2/M phase via the p53/p21/cdc2-cyclin B pathway in A549 cells.

Glucose starvation is one of the main patterns of metabolic stress in cancer. Glucose starvation can lead to reduced ATP production and enhanced ROS generation, ultimately rendering cancer cells more susceptible to cell death (Ren and Shen, 2019). AMPK is activated under stress conditions that deplete intracellular ATP and increase ADP levels, which are associated with an increased AMP/ATP ratio (Motoshima et al., 2006). AMPK suppresses protein translation and cellular growth through inhibition of the mTOR which stimulates protein synthesis, cell survival, growth, and proliferation (Sanli et al., 2014). mTOR is frequently activated in human cancer, and activated mTORC1 phosphorylates and activates downstream effectors p70S6K and the eukaryotic initiation factor 4E (eIF4E) binding protein 1 (4EBP1) to initiate protein translation/synthesis (Cornu et al., 2013; Sanli et al., 2014). AMPK has been also shown to phosphorylate p53 directly on Ser15, and phosphorylation at this site promotes p53 stabilization (Shieh et al., 1997; Jones et al., 2005). In our study, we demonstrated that PKM2-IN-1 in combination with NCT-503 caused a decrease in the intracellular ATP level when compared with PKM2-IN-1 or NCT-503 alone, thereby inducing phosphorylation of AMPK and subsequent downregulation of p-mTOR and p-p70S6K. Furthermore, combined treatment of PKM2-IN-1 and NCT-503 enhanced p-p53 (Ser15) level *versus* the single agent, thereby stabilizing the p53 level. These results suggest that PKM2-IN-1 combined with NCT-503 inhibits proliferation and induces G2/M phase cell cycle arrest through AMPK-mediated mTOR/p70S6K and p53/p21/cdc2-cyclin B pathways.

Wild-type p53 inhibits the transcription of the glucose transporter gene *GLUT1* (Schwartzenberg-Bar-Yoseph et al., 2004). Transmembrane GLUT1 is a critical rate-limiting element, that is, involved in uptake of glucose into the cells and the subsequent utilization cascade, and expression of GLUT1 is frequently elevated in numerous cancers including lung cancer (Wang et al., 2017; Cao et al., 2021; Thai et al., 2021). We found that PKM2-IN-1 in combination with NCT-503 reduced expression of GLUT1. These results suggest that combined PKM2-IN-1 and NCT-503 treatment reduces expression of GLUT1 through p53.

In the present study, we also validated that exposure of A549 cells to PKM2-IN-1 in combination with NCT-503 remarkably augmented intracellular ROS level. ROS can induce DNA damage and affect the DDR, and this response includes recognition of DNA damage, activation of check points, cell cycle arrest, and eventually final outcomes of repair, apoptosis and immune clearance (Srinivas et al., 2019). Therefore, ROS is a vital regulator of apoptosis. ATM is activated in response to DNA double-strand breaks (DSBs), and active ATM leads to phosphorylation of several target proteins regulating DNA repair, cell cycle arrest, and apoptosis such as CHK2, p53, and H2AX (Smith et al., 2020). ATM phosphorylates p53 on Ser15 (Shi and Dansen, 2020), Chk2 directly phosphorylates p53 on Ser20 (Hirao et al., 2000), resulting in p53 stabilization and activation by disrupting the interaction with MDM2. p53 activation in response to DNA damage induces cell cycle arrest, senescence, and apoptosis when DNA damage cannot be repaired (Shi and Dansen, 2020). We confirmed that the combination of PKM2-IN-1 and NCT-503 enhanced cell apoptosis when compared to PKM2-IN-1 or NCT-503 alone in A549 cells. We also found that the ROS scavenger NAC distinctly attenuated the PKM2-IN-1 in combination with NCT-503 induced apoptosis. In addition, we noted obvious increases in γ-H2AX when cells were exposed to PKM2-IN-1 in combination with NCT-503, which was suppressed by the ROS scavenger NAC. In addition, our findings showed that p-ATM and p-Chk2 were significantly increased after combined PKM2-IN-1 and NCT-503 treatment. Furthermore, we discovered that combined PKM2-IN-1 and NCT-503 treatment increased Bax expression and decreased Bcl-2 expression, and increased levels of cleaved caspase-3 and PARP. These results indicated that PKM2-IN-1 in combination with NCT-503 synergistically induced apoptosis through ROS triggered DNA damage and subsequent upregulation of pro-apoptotic Bax and downregulation of anti-apoptotic Bcl-2/activated caspase-3 led to PARP cleavage signaling pathway. Therefore, the above results suggest that the effect of PKM2-IN-1 in combination with NCT-503 on cell cycle arrest and apoptotic induction may be the main mechanism that attenuates tumor growth.

In the A549 xenograft model, the PKM2-IN-1 and NCT-503 combined treatment significantly inhibited tumor growth compared with PKM2-IN-1 or NCT-503 alone. Furthermore, inhibition of tumor cell proliferation (Ki67) was observed in combination treatment PKM2-IN-1 and NCT-503 compared with each single agent treatment and control group. The expression of Ki67 is strongly associated with tumor cell proliferation and is widely used in routine pathology (Li et al., 2015). Taken together, these findings demonstrate that PKM2-IN-1 in combination with NCT-503 can effectively inhibit proliferation and growth of lung cancer cells *in vitro* and *in vivo*.

## Conclusion

Our studies identified PKM2-IN-1 in combination with NCT-503 as a new treatment of cancer therapy that induces cell apoptosis by the Bcl-2/caspase-3/PARP pathway and promotes G2/M phase arrest and inhibits proliferation by p53/p21/cdc2-cyclin B and mTOR/p70S6K pathways through the ROS-induced DNA damage and AMPK dependent pathways in A549 cells. Furthermore, combined treatment inhibited expression of GLUT1 level. These findings provide an insight into exploring the tumor therapeutic efficacy of PKM2-IN-1 in combination with NCT-503 and will be useful for new anti-cancer drug combination strategies.

## Data Availability

The original contributions presented in the study are included in the article/Supplementary Material, further inquiries can be directed to the corresponding authors.
